# Glass in the Airways: A Bronchoscopic Challenge

**DOI:** 10.7759/cureus.53344

**Published:** 2024-01-31

**Authors:** Sai Doppalapudi, Ked Fortuzi, Abeer Qasim, Diaz Saez Yordanka, Misbahuddin Khaja

**Affiliations:** 1 Pulmonary and Critical Care Medicine, BronxCare Hospital, New York City, USA; 2 Pulmonary Medicine, BronxCare Hospital, New York City, USA; 3 Internal Medicine, BronxCare Hospital, New York City, USA

**Keywords:** chronic cough, rigid bronchoscopy, aspiration, bronchoscopy, foreign body aspiration

## Abstract

Foreign body aspiration (FBA) in adults is indeed a significant medical concern, albeit less common than in children. The increase in incidence with advancing age can be attributed to factors such as a decline in mental status and impairment of the swallowing reflex, which is more prevalent in the elderly population. The symptoms of FBA are highly variable, ranging from severe, acute asphyxiation, which may or may not involve complete airway obstruction, to more subtle signs like coughing, shortness of breath (dyspnea), choking, or fever. These varied presentations, coupled with the fact that many other medical conditions can mimic the respiratory symptoms seen in FBA, make diagnosis challenging. A high index of suspicion is often required, especially in cases where the patient's history does not clearly point toward aspiration. Immediate management focuses on supporting the airway, which is crucial given the potential for severe obstruction. Radiographic imaging plays a key role in localizing the foreign body, which is vital for planning its removal. Bronchoscopy, particularly flexible bronchoscopy, is the cornerstone of both diagnosis and treatment. This technique allows for direct visualization of the airways, localization of the foreign body, and its subsequent removal. This is crucial to avoid long-term complications, which can arise if the foreign body is not promptly and effectively removed. In this case report, we present a 64-year-old female patient who was found to have a foreign object positioned in the right lower lobe of the lungs that was removed via flexible bronchoscopy.

## Introduction

Foreign body aspiration (FBA) in adults can potentially be a life-threatening event often manifesting as a choking episode accompanied by sustained coughing and dyspnea. The initial episode can rarely go unnoticed, and its sequela mimics chronic conditions such as asthma, chronic obstructive pulmonary disease, or infectious pneumonia [1]. Aspiration in adults usually occurs in those with an underlying neurological impairment, substance use disorder, psychiatric conditions, or traumatic head injury. Bronchial foreign bodies commonly find their resting place in the right main bronchus, primarily due to its more vertical orientation relative to the trachea, branching at an angle of about 25 degrees. Additionally, the location of the carina to the left of the midline contributes to this tendency [2]. In this case report, we present a 64-year-old female patient who was found to have a foreign object positioned in the right lower lobe of the lungs that was removed via flexible bronchoscopy.

## Case presentation

A 64-year-old female with a history of schizophrenia, polysubstance use disorder (alcohol, cocaine, tobacco, cannabis), human immunodeficiency virus (HIV), diabetes mellitus (DM), and chronic obstructive pulmonary disease (COPD) presented to the emergency department with complaints of chills, generalized body ache, and headaches. She was referred from a mental health facility after observing her oxygen saturation drop to 80% on room air. Upon arrival at the emergency department, she was noted to be hypoxemic and needed supplemental oxygen but with no signs of respiratory distress, including no use of accessory muscles for breathing, and was able to speak in full sentences. She denied hemoptysis or chest pain. Her vital signs were otherwise stable with a blood pressure of 127/78 mmHg, a pulse rate of 62 beats per minute, and a body temperature of 97.9°F. Her physical examination was remarkable for rhonchi heard in the right hemithorax. Laboratory findings were significant for anemia with a hemoglobin of 11.1 g/dl, hyponatremia (sodium of 131 mEq/L), acute kidney injury (creatinine of 1.7 mg/dL), and elevated D-dimers (344 ng/mL). Other laboratory findings are shown in Table 1.

**Table 1:** Lab results from the time of admission

Test name	Results	Normal range
White blood cell count	7.1 k/ul	4.8-10.8 k/ul
Red blood cell count	3.79 MIL/ul	4.00-5.20 MIL/ul
Hemoglobin	11.1 g/dl	12.0-16.0 g/dl
Hematocrit, whole blood	32.8%	42.0%-51.0%
Mean corpuscular volume	86.5 fL	80.0-96.0 fL
Mean corpuscular hemoglobin	29.1 pg	27.0-33.0 pg
Mean corpuscular hemoglobin concentration	33.7 g/pg	33.0-36.0 g/dl
Mean platelet volume	8.9 fL	8.0-12.0 fL
Red cell distribution width	14.8%	10.5%-14.5%
Platelet	163 k/ul	150-400 k/ul
Neutrophil %	71.5%	40.0%-70.0%
Neutrophil count	5.1 k/ul	1.5-8.0 k/ul
Lactic acid level	1.4 mmoles/L	0.5-1.6 mmoles/L
Sodium, serum	131 mEq/L	135-145 mEq/L
Potassium, serum	3.8 mEq/L	3.5-5.0 mEq/L
Blood urea nitrogen, serum	22.0 mg/dL	6.0-20.0 mg/dL
Creatinine, serum	1.7 mg/dL	0.5-1.5 mg/dL
D-dimer assay, plasma	344 ng/mL	0-230 ng/mL
International normalized ratio	1.07	0.85-1.29

**Figure 1:**
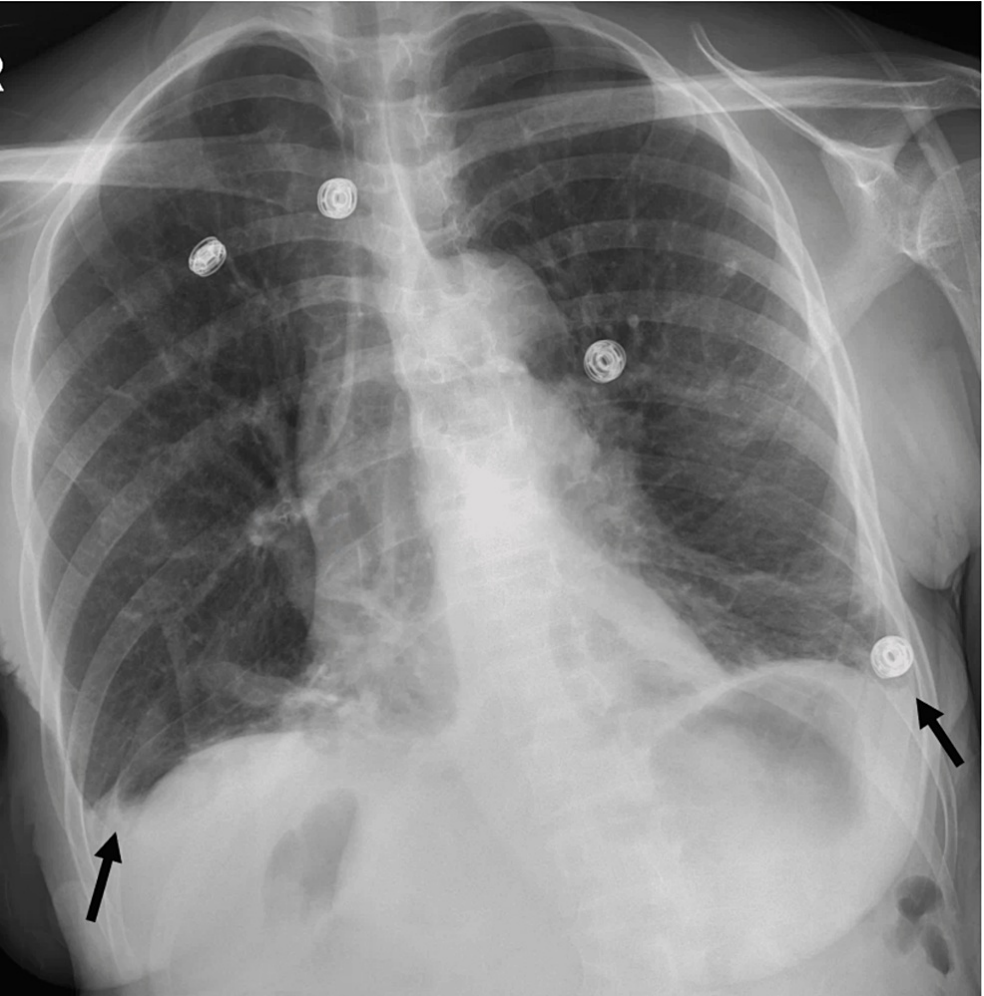
Chest X-Ray showing new small bilateral pleural effusions

She later underwent a chest X-ray, which revealed small bilateral pleural effusions, mild bibasilar infiltrate/atelectasis, and stable diffuse emphysematous changes (Figure 1).

**Figure 2:**
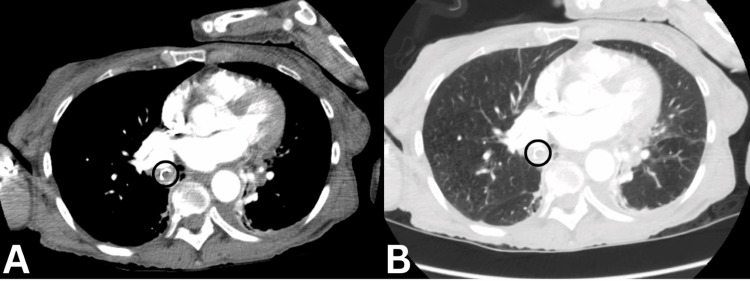
Computed tomography scan (axial view) showing 9-mm radiopaque body within the right lower lobe: (A) mediastinum window and (B) lung window

**Figure 3:**
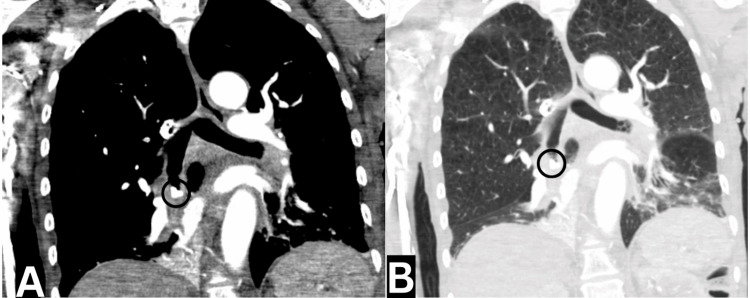
Computed tomography scan (coronal View) showing near-complete opacification of the right lower lobe: (A) mediastinum window and (B) lung window

Additionally, a CT angiogram of the chest was performed revealing a peribronchial and bibasilar pleural-based consolidations, with the right side more affected than the left, suggestive of an infectious or inflammatory process/pneumonitis. Furthermore, a 0.9 mm curvilinear radiopaque body was identified within the right lower lobe bronchus, accompanied by adjacent debris (Figure 2). There was near-complete opacification of the right lower lobe. Other findings included upper lobe-predominant centrilobular emphysema and a 5-mm pleural-based pulmonary nodule in the right upper lobe (Figure 3).

The patient was admitted to ICU due to concerns of hypoxemia in the setting of suspected FBA. Based on the presumptive diagnosis of post-obstructive pneumonia, she was initiated on appropriate broad-spectrum antibiotics with vancomycin, zosyn, and azithromycin. She was then taken to the operating room and underwent flexible bronchoscopy (FOB) under general anesthesia. The foreign body, which was measured to be 1 cm, had a larger diameter than the endotracheal tube and would not be able to be passed through it during retrieval. An 8-mm endotracheal tube (ETT) was inserted and in anticipation of difficult retrieval, it was agreed with the anesthesiologist that the endotracheal tube would have to come out together with the foreign body and then reintubate immediately after to secure the airway.

**Figure 4:**
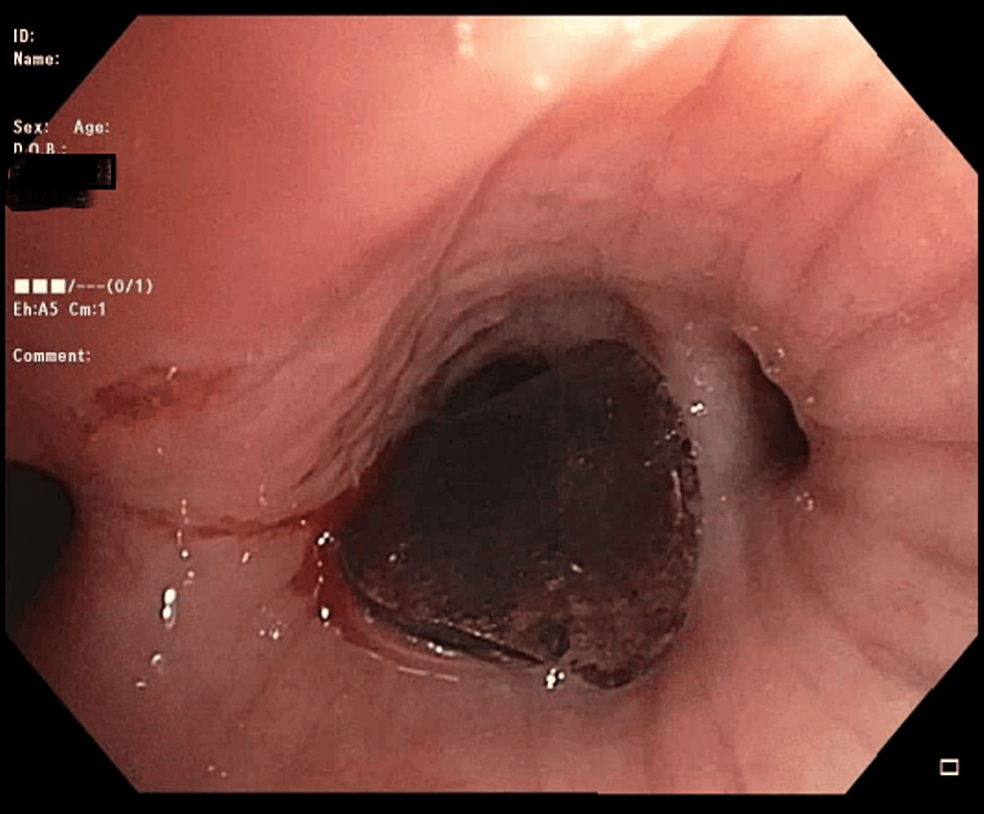
A close-up of the foreign body obstructing the right lower lobe

The procedure went accordingly, and a complete tracheobronchial tree was visualized. Carina was sharp with no lesions, and there was no excessive dynamic collapse or concern for tracheobronchomalacia. A quick inspection of the left side was performed, and no anomalies were visualized. The FOB was passed through the main right bronchus, the right upper lobe was visualized, and all three segments were patent. FOB was then advanced to the bronchus intermedius, and the foreign body was visualized lodged in the right lower lobe bronchus partially occluding its opening to the superior segment (Figure 4).

**Figure 5:**
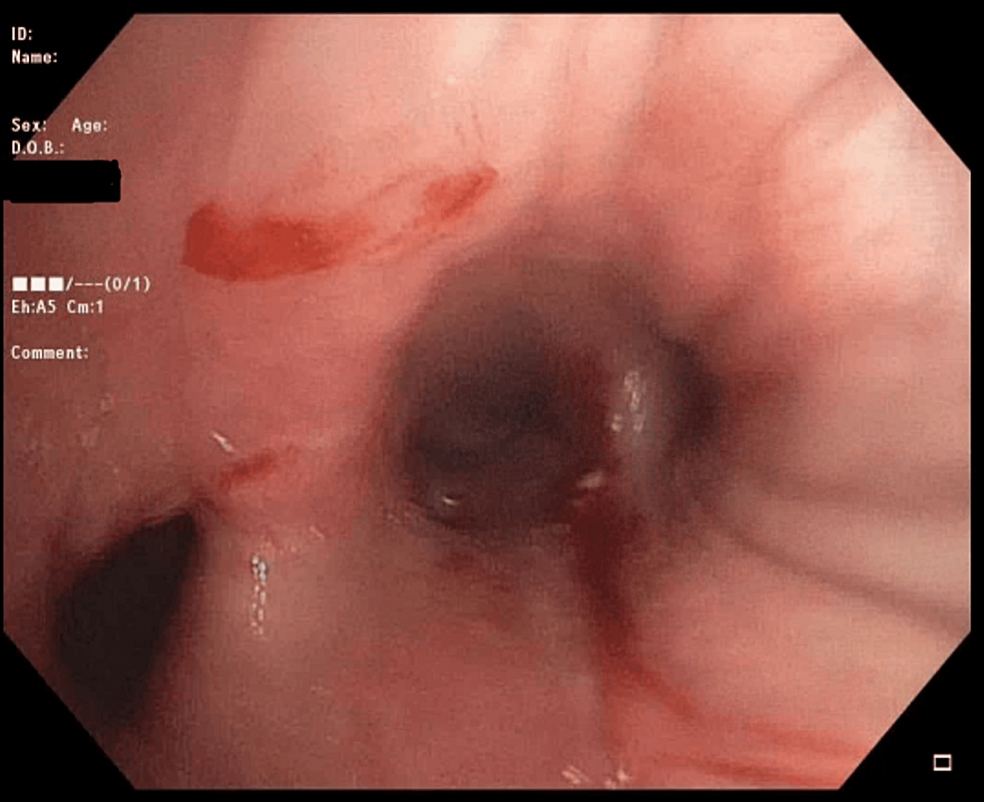
A close-up visualizing the right middle lobe, right lower lobe, and its superior segment after retrieval of the foreign body

Using a grasping forceps, the foreign body was carefully retrieved and brought up to the trachea at the end of the ETT. As expected, the diameter of the ETT was narrower than the aspirated object, and as planned, she was extubated with both the ETT and the foreign body. After reintubation, a final inspection was performed. There was no bleeding or lacerations of the airway, and moderate mucopurulent secretions were noted in the right lower lobe (Figure 5). Bronchoalveolar lavage (BAL) was done from the posterior segment of the right lower lobe and sent for aerobic respiratory culture. The patient tolerated the procedure well, and post-procedure imaging showed improved aeration along the right lower lobe. It was later found that the piece of glass was part of a “crack pipe” that the patient was using and accidentally aspirated.

## Discussion

Aspiration is a relatively common event that is typically well-tolerated. Numerous studies indicate that virtually all healthy persons aspirate, but this is usually inconsequential. FBA, more apparent in children than in adults, is usually recognized from patients’ history. In adults, the peak incidence is in the sixth decade when impairment of normal protective mechanisms is common. It is associated with neurological disorders, trauma with loss of consciousness, sedatives or alcohol use, and poor dentition. The penetration syndrome, defined as the sudden onset of choking and intractable cough after aspirating in a foreign body, with or without vomiting, is often followed by persistent cough, fever, chest pain, dyspnea, and wheezing. Objects can enter both the respiratory tract and the digestive tract through the mouth and nose. However, when an object enters the respiratory tract, it is specifically referred to as aspiration [3]. Subsequently, the foreign object may become trapped in the trachea or go deeper into the respiratory system, namely within a bronchus and its segments. Irrespective of the nature of the object, any aspiration can pose a serious risk to life and necessitates prompt identification and intervention to reduce the likelihood of consequences [4]. The signs and symptoms of FBA differ depending on the location of the blockage, the size and composition of the object, and the degree of obstruction.

About 20% of foreign objects get stuck in the upper airway, while 80% get stuck in a bronchus [4]. Stridor is commonly observed in cases when foreign bodies are located above the larynx, whereas wheezing is typically associated with objects situated below the larynx. Foreign objects located above the vocal cords frequently manifest as dysphagia, odynophagia, and severe sialorrhea. Subglottic foreign bodies frequently manifest as dysphonia and dyspnea, accompanied by pain. In children who are unable to communicate or indicate if they have swallowed a foreign object, an elevated respiration rate may be the sole indication of FBA. In cases where the foreign object does not result in significant blockage, individuals may exhibit symptoms such as persistent cough, uneven breath sounds on examination, or recurring pneumonia affecting a particular section of the lung. FBA can lead to recurrent pneumonia in adults, with the right lower lobe of the lung being the primary affected region [5,6]. This is because the right major bronchus has a wider and steeper architecture compared to the left main bronchus, which makes it easier for things to enter.

Chest radiography has poor sensitivity for the detection of foreign bodies with a sensitivity of approximately 70%-80% in adults. Computed tomography has a better sensitivity of 99% for detection with a better resolution and can better help in locating the object in preparation for endoscopic intervention [6]. The most reliable CT finding of an aspirated foreign body is its demonstration within the lumen of the tracheobronchial tree [7]. Associated features are usually secondary parenchymal changes in the affected lobes and reactive mediastinal findings, including hilar lymphadenopathy and bronchial thickening. The visibility of an object is influenced by various elements, including the object's material, size, anatomical placement, surrounding structures, and the patient's body habitus.

Bronchoscopy is the method of choice for foreign body retrieval from airways [8]. The choice of flexible versus rigid bronchoscopy is often operator and institution-dependent. The solid steel barrel of the rigid bronchoscope protects and reinforces the trachea, larynx, and vocal cords, shielding such structures from injury when removing sharp or irregularly shaped objects. The large bore lumen of the rigid bronchoscope is also beneficial in allowing adequate ventilation even when moving large objects with the potential to obstruct conventional endotracheal tubes. Additionally, the greater luminal dimension allows for the use of larger, rigid grasping forceps, retrieval baskets, and suction channels simultaneously. Flexible bronchoscopes can also be passed through the lumen of the rigid bronchoscopy when angled manipulation is required [9,10].

Flexible bronchoscopy can be employed for extraction in cases where distal access is required, and the operator has expertise in this technique [11]. Possible benefits include the avoidance of general anesthesia and the capability to access subsegmental bronchi, which are narrower and located deeper in the respiratory system compared to the major bronchi. The primary drawback of employing a flexible scope is the potential for exacerbating the displacement of the object and resulting in obstruction of the airway. Bronchoscopy has a success rate of roughly 95% in removing foreign bodies, with a complication rate of only 1% [12,13].

## Conclusions

FBA is often a concern for the pediatric population compared to adults. Nevertheless, it can potentially be a life-threatening event that can manifest as a choking episode accompanied by sustained coughing and dyspnea. However, aspiration goes unnoticed often until patients suffer from its sequela. We describe a case where a patient presented with a sole symptom of hypoxemia that led to the discovery of a foreign body that was aspirated years prior. It is important to keep in mind that foreign body aspiration can go unrecognized especially in adults until the patient develops sequela to this culprit event.
